# Imported malaria into Australia: surveillance insights and opportunities

**DOI:** 10.1093/jtm/taad164

**Published:** 2023-12-21

**Authors:** Asma Sohail, Alyssa Barry, Sarah Auburn, Qin Cheng, Colleen L Lau, Rogan Lee, Ric N Price, Luis Furuya-Kanamori, Paolo Bareng, Sarah L McGuinness, Karin Leder

**Affiliations:** School of Public Health and Preventive Medicine, Monash University, Melbourne 3004, Australia; Department of Infectious Diseases, Grampians Health, Ballarat 3350, Australia; Institute for Physical and Mental Health and Clinical Translation (IMPACT) and School of Medicine, Deakin University, Geelong 3220, Australia; Disease Elimination Program, Burnet Institute, Melbourne 3004, Australia; Global and Tropical Health Division, Menzies School of Health Research, Charles Darwin University, Darwin 0800, Australia; Drug Resistance and Diagnostics, Australian Defence Force Malaria and Infectious Disease Institute, Brisbane 4051, Australia; School of Public Health, Faculty of Medicine, The University of Queensland, Herston 4006, Australia; Parasitology Unit, Institute of Clinical Pathology and Medical Research, Sydney 2145, Australia; Global and Tropical Health Division, Menzies School of Health Research, Charles Darwin University, Darwin 0800, Australia; Centre for Tropical Medicine and Global Health, Nuffield Department of Clinical Medicine, University of Oxford, Oxford OX1 2JD, UK; School of Public Health, Faculty of Medicine, The University of Queensland, Herston 4006, Australia; Institute for Physical and Mental Health and Clinical Translation (IMPACT) and School of Medicine, Deakin University, Geelong 3220, Australia; School of Public Health and Preventive Medicine, Monash University, Melbourne 3004, Australia; Department of Infectious Diseases, Alfred Health, Melbourne 3004, Australia; School of Public Health and Preventive Medicine, Monash University, Melbourne 3004, Australia; Victorian Infectious Diseases Service, Melbourne Health, Melbourne 3052, Australia

**Keywords:** Infectious disease, notifiable disease, epidemiology, genomics, genetics, molecular surveillance, travel, Plasmodium

## Abstract

**Background:**

Malaria continues to pose a significant burden in endemic countries, many of which lack access to molecular surveillance. Insights from malaria cases in travellers returning to non-endemic areas can provide valuable data to inform endemic country programmes. To evaluate the potential for novel global insights into malaria, we examined epidemiological and molecular data from imported malaria cases to Australia.

**Methods:**

We analysed malaria cases reported in Australia from 2012 to 2022 using National Notifiable Disease Surveillance System data. Molecular data on imported malaria cases were obtained from literature searches.

**Results:**

Between 2012 and 2022, 3204 malaria cases were reported in Australia. Most cases (69%) were male and 44% occurred in young adults aged 20–39 years. Incidence rates initially declined between 2012 and 2015, then increased until 2019. During 2012–2019, the incidence in travellers ranged from 1.34 to 7.71 per 100 000 trips. Cases were primarily acquired in Sub-Saharan Africa (*n* = 1433; 45%), Oceania (*n* = 569; 18%) and Southern and Central Asia (*n* = 367; 12%). The most common countries of acquisition were Papua New Guinea (*n* = 474) and India (*n* = 277). *Plasmodium falciparum* accounted for 58% (1871/3204) of cases and was predominantly acquired in Sub-Saharan Africa, and *Plasmodium vivax* accounted for 32% (1016/3204), predominantly from Oceania and Asia. Molecular studies of imported malaria cases to Australia identified genetic mutations and deletions associated with drug resistance and false-negative rapid diagnostic test results, and led to the establishment of reference genomes for *P. vivax* and *Plasmodium malariae*.

**Conclusions:**

Our analysis highlights the continuing burden of imported malaria into Australia. Molecular studies have offered valuable insights into drug resistance and diagnostic limitations, and established reference genomes. Integrating molecular data into national surveillance systems could provide important infectious disease intelligence to optimize treatment guidelines for returning travellers and support endemic country surveillance programmes.

## Background

Malaria remains a significant global health challenge. In 2021, an estimated 247 million malaria cases and 619 000 deaths were reported worldwide, with a disproportionate burden in sub-Saharan Africa.^1^ In the last two decades, the number of malaria-endemic countries has fallen^2^ and global malaria incidence and death rates have declined, but since 2015, progress towards elimination goals has slowed and even reversed in some locations.^3^^,^^4^ The situation has been further confounded by healthcare disruptions during the COVID-19 pandemic (2020–2021) which resulted in ~13 million additional malaria cases and 63 000 more malaria deaths.^1^^,^^5^ Ongoing challenges include insecticide resistance in malaria vectors,^6^^,^^7^ geographical vector expansion due to climate change and adaption to urban environments,^8^^,^^9^ the emergence and spread of antimalarial drug resistance^10^^,^^11^ and rapid diagnostic tests (RDTs) failures.^12^^,^^13^

Globally, travel-related malaria surveillance data are primarily derived from North American and European travellers, with most cases acquired in sub-Saharan Africa.^14^^,^^15^ Endemic transmission of malaria was eliminated from Australia in 1981, but the risk of reintroduction remains due to the presence of *Anopheles* mosquito vectors in the country’s tropical north.^16^ Previous reports indicate that Australia receives an average of 220–600 malaria cases per year.^15^^,^^17^ Australia’s geographical location and migrant diversity affect malaria acquisition patterns, with a higher proportion of cases acquired in Oceania and Asia compared with other non-endemic nations.^15^^,^^17^^,^^18^ Therefore, data on imported infections to Australia can help bridge gaps in global malaria surveillance.

Epidemiological and molecular surveillance of imported malaria in non-endemic countries informs pre-travel advice, guides management of ill returned travellers and facilitates monitoring of dynamic epidemiological patterns in endemic countries.^15^^,^^19^^,^^20^ Malaria parasites exhibit extensive genetic diversity that drives critical adaptations, such as antimalarial drug resistance, immune evasion and gene deletions that limit detection by widely used RDTs.^21^^,^^22^ Molecular techniques can capture parasite genetic information at varying scales, ranging from targeted regions to whole genome sequencing (WGS).^23^^,^^24^ These include capillary-based sequencing, quantitative Polymerase Chain Reaction (qPCR) and Next Generation sequencing (e.g. Illumina, Oxford Nanopore Technologies, PacBio).^23^^,^^25–28^ Although *Plasmodium vivax* WGS remains challenging, *Plasmodium falciparum* genomes can be readily sequenced using various platforms.^21^^,^^29–31^ Whilst amplicon sequencing remains preferable for endemic surveillance due to its lower cost and scalability, WGS is used in research for biomarker discovery, genome-wide association and discovery projects.^32–35^

In countries with advanced laboratory and bioinformatics capacity, insights from molecular testing of imported malaria cases could potentially aid in informing risk assessment, antimalarial treatment guidelines and potentially even acute case management for the returning traveller. Additionally, it has the potential to provide important intelligence to support endemic country surveillance programmes.

We aimed to describe the epidemiological and molecular characteristics of imported malaria to Australia through analysis of notifiable diseases data and molecular studies.

## Methods

### Data sources

#### Malaria cases

We collated data on notified malaria cases from the National Notifiable Diseases Surveillance System (NNDSS) between 1 January 2012 and 31 December 2022.

The NNDSS is an Australian passive surveillance system established in 1991, capturing notifiable disease data from all Australian jurisdictions.^36^ Each jurisdiction collects data from doctors and/or laboratories and forwards de-identified data for diseases on the National Notifiable Disease List (NNDL), which includes malaria, onto the NNDSS. We obtained NNDSS data for this study from the Australian Government’s Office of Health Protection in June 2023.

#### Population and traveller movement estimates

National and jurisdictional resident population estimates on 30 June each year and Australian traveller movement data (2012–2022) were obtained from the Australian Bureau of Statistics (ABS).^37^^,^^38^ Traveller movements are defined not by individual travellers or trips but rather by international border crossings categorized as short term (<12 months overseas) resident departures (STRD; 2012–2017) and returns (STRR; 2017–2022), short term (intending to visit Australia for < 12 months) visitor arrivals (STVA), long term (>12 months overseas) resident returns (LTRR), long term (intending to stay in Australia for > 12 months) visitor arrivals (LTVA) and permanent arrivals. These data encompass leisure, business, visiting friends and relatives (VFR) travel, foreign visitor arrivals and migrant travel.

Countries were grouped into geographical regions using the Standard Australian Classification of Countries (SACC) 2016 (see Supplementary Material).^39^

#### Surveillance and molecular data and capacity

We searched PubMed and Google Scholar for additional epidemiological and molecular data on imported malaria cases. Additional data were supplemented by contributing authors, with details on existing malaria molecular research capacity in Australia (see Supplementary Material).

### Epidemiological analysis

The total and annual incidence rates of notified cases were calculated nationally and by jurisdiction. Incidence rates were derived from all notified cases (the numerator), with denominators taken from ABS resident population estimates on 30 June of each study year (‘population incidence’) and ABS traveller movement data (‘incidence by traveller movement’). The number of traveller movements per jurisdiction was based on travellers indicated ‘state of residence’ on the incoming passenger card that all international arrivals complete when entering Australia.

Data were analysed using STATA version 15 (StataCorp. 2017. *Stata Statistical Software: Release 15*. College Station, TX: StataCorp LLC).

### Ethics approval

The project was approved by the Monash University Human Research Ethics Committee (MUHREC; project #28955) and Communicable Disease Network Australia (CDNA) jurisdictional members.

## Results

### All malaria cases notified to the NNDSS

**Figure 1:**
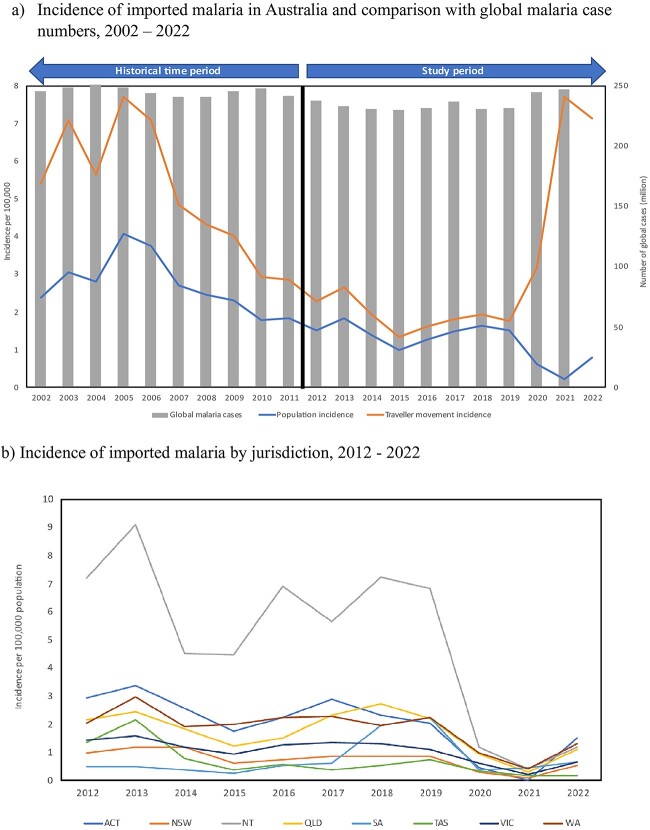
Incidence of imported malaria cases into Australia. (a) Incidence of imported malaria in Australia and comparison with global malaria case numbers, 2002–2022. Population and traveller movement incidence follow the primary axis. Global malaria cases follow the secondary axis. Aggregate data on malaria notifications in Australia from 2002–2011 obtained from NNDSS Annual Reports, 2003–2012. Incidence rates calculated using all notified cases as the numerator, with denominators taken from ABS resident population estimates on 30 June of each study year (‘population incidence’) and ABS traveller movement data (‘incidence by traveller movement’). Data on global malaria cases obtained from the World Health Organisation’s World Malaria Report, 2022. Data on cases for 2022 not available at time of writing. (b) Incidence of imported malaria by jurisdiction, 2012–2022. Abbreviations: ACT: Australian Capital Territory; NSW: New South Wales; NT: Northern Territory; QLD: Queensland; SA: South Australia; TAS: Tasmania; VIC: Victoria; WA: Western Australia.

From January 2012 to December 2022, a total of 3204 malaria cases were notified to the NNDSS, with a median of 324 (range: 59–422) cases. The annual population incidence declined from 1.52 [95%CI: 1.37–1.69] per 100 000 person-years (py) in 2012 to 0.98 [95% CI: 0.87–1.12] per 100 000 py in 2015. However, between 2015 and 2019 the annual population incidence increased (Figure 1a). The incidence in travellers ranged from 1.34 [95% CI: 1.17–1.52] to 2.65 [95% CI: 2.40–2.91] notifications per 100 000 annual traveller movements (Figure 1a; Supplementary Material, Table S1). During the COVID-19 pandemic, annual incidence in travellers increased to 3.17 [95% CI: 2.70–3.71] per 100 000 traveller movements in 2020 and 7.71 [95% CI: 5.87–9.95] per 100 000 traveller movements in 2021. Traveller movements increased in 2022, associated with a slight decrease in the incidence to 7.15 [95% CI: 6.21–8.20] per 100 000 (Figure 1a; Supplementary Material, Table S1).

Overall, 2196 (69%) of cases occurred in males and 1393 (44%) in young adults aged 20–39 years; 85 (3%) cases were reported in children < 5 years (Table 1). The greatest number of notifications were reported in Queensland (29%; *n* = 917) (Table 1), whereas the highest notification incidence was reported from the Northern Territory (4.98 [95% CI: 2.80–8.51] per 100 000py (Figure 1b).

**Table 1:** Characteristics of imported malaria cases, Australia 2012–2022

Characteristic	All cases *n*, (%)	*P. falciparum* *n*, (%)	*P. vivax* *n*, (%)	*P. ovale* *n*, (%)	*P. malariae* *n*, (%)	*P. knowlesi* *n*, (%)	Mixed species *n*, (%)	Species unspecified *n*, (%)
Total cases, *n*	3204	1840	996	167	98	2	34	67
Age group, *n* (%)^a^ < 5 years 5–19 years 20–39 years 40–59 years ≥60 years	85 (3) 514(16) 1393 (43) 946 (30) 264 (8)	59 (3) 310 (17) 777 (42) 581 (32) 112 (6)	10 (1) 151 (15) 453 (46) 261 (26) 121 (12)	9 (6) 14 (8) 81 (49) 47 (28) 16 (9)	2 (2) 21 (21) 44 (45) 21 (21) 10 (11)	1 (50) 1 (50)	2 (6) 8 (24) 14 (41) 9 (26) 1 (3)	3 (5) 10 (15) 23 (34) 26 (39) 4 (6)
Male, *n* (%)	2196 (69)	1245 (68)	693 (70)	109 (65)	68 (69)	2 (100)	28 (82)	51 (76)
Jurisdiction, *n* (%) Australian Capital Territory New South Wales Northern Territory Queensland South Australia Tasmania Victoria Western Australia	90 (3) 633 (20) 134 (4) 917 (29) 147 (5) 40 (1) 720 (22) 523 (16)	54 (3) 335 (18) 81 (5) 472 (26) 110 (6) 25 (1) 392 (21) 371 (20)	29 (3) 208 (21) 39 (4) 360 (36) 28 (3) 3 (<1) 243 (24) 86 (9)	3 (2) 47 (28) 7 (4) 41 (24) 3(2) 40 (24) 26 (16)	1 (1) 17 (17) 3 (3) 32 (33) 3 (3) 27 (28) 15 (15)	1 (50) 1 (50)	1 (3) 4 (12) 2 (6) 1 (3) 3 (9) 10 (29) 13 (38)	2 (3) 26 (39) 8 (12) 2 (3) 9 (14) 8 (12) 11 (17)
Region of acquisition, *n* (%) Sub-Saharan Africa Oceania Southern Central Asia North Africa and the Middle East Southeast Asia Northeast Asia Northwest Europe Southern and Eastern Europe The Americas Unknown region/missing data	1433 (45) 569 (18) 367 (11) 348 (11) 194 (6) 8 (<1) 1 (<1) 1 (<1) 17 (<1) 266 (8)	1170 (64) 167 (9) 15 (<1) 275 (15) 57 (3) 1 (<1) 3 (<1) 152 (8)	52 (5) 383 (38) 337 (34) 16 (2) 117 (12) 6 (<1) 1 (<1) 13 (1) 71 (7)	104 (62) 3 (2) 4 (3) 29 (17) 7 (4) 1 (<1) 19 (11)	64 (65) 5 (5) 2 (2) 12 (12) 3 (3) 1 (1) 1 (1) 10 (11)	2 (100)	15 (45) 3 (9) 3 (9) 5 (14) 5 (14) 3 (9)	28 (42) 8 (12) 6 (9) 11 (17) 3 (5) 10 (15)

^a^Data unavailable for two cases.

**Figure 2:**
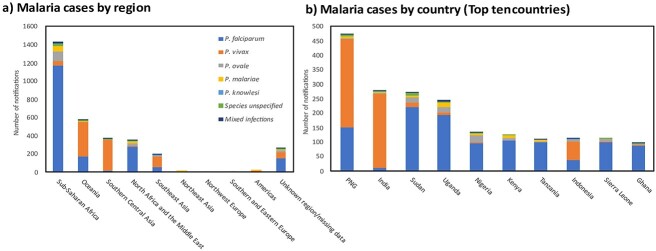
Geographic origins of malaria cases imported into Australia from 2012–2022. Malaria case numbers for each *Plasmodium* species stratified by (A) Region and (B) Country. Abbreviations: PNG: Papua New Guinea. Countries shown include the top 10 countries of acquisition by notification numbers. Sudan and South Sudan are classified under North Africa and the Middle East.

Malaria was acquired in Sub-Saharan Africa in 1433 (45%) of cases, with 569 (18%) cases reported in Oceania and 367 (12%) in Southern and Central Asia (Table 1, Figure 2a; Supplementary material, Figure S1). The most common countries of acquisition were Papua New Guinea (PNG) (15%, *n* = 474) and India (9%, *n* = 277) (Figure 2b). PNG was the most common country of acquisition for cases reported from Queensland and the Australian Capital Territory (ACT), whereas India was the most common country of acquisition for cases reported from New South Wales and Victoria (Supplementary Material, Table S2).

### Imported malaria cases to Australia by *Plasmodium* species

**Figure 3:**
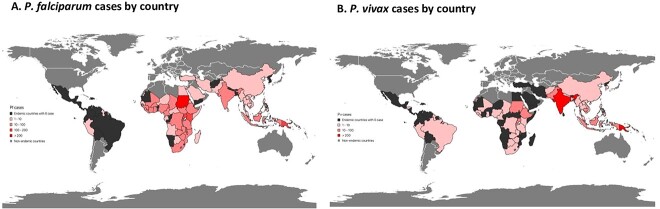
Distribution of origins of *P. falciparum* and *P. vivax* cases imported in Australia from 2012–2022. Malaria case numbers are indicated by shading for (A) *P. falciparum* cases and (B) *P. vivax* cases.

A single *Plasmodium* species was reported in 3103 (97%) of cases, with *P. falciparum* accounting for 59% (*n* = 1840), *P. vivax* 32% (*n* = 996), *Plasmodium ovale* 5% (*n* = 167) and *Plasmodium malariae* 3% (*n* = 98) (Table 1; Figure 2). *Plasmodium falciparum* was most commonly acquired in Sub-Saharan Africa (1170/1840; 64%), particularly from Uganda (*n* = 194; 11%) and Nigeria (*n* = 95; 5%) (Table 1, Figures 2 and 3). Conversely, *P. vivax* was mostly commonly acquired in Oceania (383/996; 39%) or Southern and Central Asia (337/996; 34%), primarily from PNG (*n* = 305; 31%) or India (*n* = 255; 26%) (Table 1, Figures 2 and 3). Sub-Saharan Africa was the most common region of acquisition for *P. ovale* and *P. malariae* (Table 1, Figure 2a). Only two cases of *Plasmodium knowlesi* were recorded, one from the Philippines and one from Indonesia.

Overall, mixed *Plasmodium* infection were reported in 34 (1%) cases of which 17 were *P. falciparum* and *P. vivax*, 9 *P. falciparum* and *P. ovale*, 5 *P. falciparum* and *P. malariae*, 2 *P. vivax and P. ovale* and 1 of *P. vivax* and *P. malariae* (Table 1).

### Molecular studies of imported malaria to Australia

We identified six studies detailing molecular insights from cases imported to Australia (Table 2). Two studies analysed samples from imported *P. falciparum* cases. One screened samples for *pfhrp2* deletions,^40^ linked to false negative results on some RDTs. *Pfhrp2 *deletion rates exceeded 10% in cases from Nigeria, Sudan and South Sudan. The other screened samples for mutations in the *Pfkelch13* gene,^41^ associated with artemisinin resistance and delayed parasite clearance. This study reported the first detection of the *kelch13* C580Y mutation in a case acquired in PNG; the patient did not experience treatment failure following artemisinin combination therapy.^41^ The next study sequenced imported *P. vivax* case samples for molecular markers of drug resistance, identifying mutations associated with resistance to chloroquine (in *Pvmdr1* and *Pvcrt-o* genes) and sulphadoxine-pyrimethamine (*Pvdhfr *and *Pvdhps* genes).^42^ Another study employed WGS to demonstrate clonal *P. malariae* recrudescence following artemisinin combination therapy in an imported case from Uganda.^43^ The remaining two studies established referenced genomes for *P. vivax* and *P. malariae* in cases imported from Indonesia and Uganda, respectively.^44^^,^^45^

**Table 2:** Notable molecular studies on imported malaria from Australia

Author	Study aim	Number of samples (*n*)	Data collection period	*Plasmodium* Species	Region or country of malaria acquisition	Key findings/outcomes
Prosser *et al.*^40^	To identify evidence for *pfhrp2* and *pfhrp3* deletions in imported *P. falciparum* cases	210 samples (210 cases)	2010–2018	*P. falciparum*	Nigeria, Sudan, Kenya, Ghana, other African countries	Identified *pfhrp2* and *pfhrp3* deletions in cases from 12 countries, with *pfhrp2*-deletion rates exceeding 10% in cases from Nigeria, Sudan and South Sudan.
Prosser *et al.*^41^	To characterize the *Pfkelch13 *propeller domains of imported *P*. *falciparum* cases	153 samples (140 cases)	2010–2016	*P. falciparum*	Papua New Guinea, Nigeria, Sudan, Kenya, Ghana, other African countries, India, Indonesia	First detection of the *kelch13* C580Y mutation in a case from Papua New Guinea. Low level of *Pfkelch13* propeller domain diversity overall, with no coding mutations observed in imported cases from Africa.
Noisang et al.^42^	To detect the prevalence of molecular resistance markers for chloroquine, sulfadoxine and pyrimethamine in *P. vivax*-infected travellers	120 samples^a^	2008–2018	*P. vivax*	India, PNG, Pakistan, Indonesia, Thailand, Solomon Islands, South Korea, Cambodia	Characterized mutations in *P. vivax* infections of imported cases. Detected mutations in the *Pvmdr1*, *Pvcrt-o*, *Pvdhfr and* *Pvdhps* genes. Demonstrated an increase in resistance markers seen in cases from all countries.
Auburn *et al.*^44^	To establish a reference genome for *P. vivax* in order to address the research need for a comprehensive *vivax* genome	Three samples (one case)	2012	*P. vivax*	Papua Indonesia	Assembly and annotation of a new reference genomic sequence for *P. vivax*, *PvP01*.
Rutledge *et al.*^43^	To report a case of a *P. malariae* infection that resulted in recrudescence months after treatment with artemether/lumefantrine	Two samples (one case)	2015	*P. malariae*	Uganda	Reported a case of *P. malariae* recrudescence following directly observed artemether/lumefantrine therapy in an imported case from Uganda. Whole-genome sequencing revealed that the recrudescent isolate was a single clone present at low density in the initial polyclonal infection.
Rutledge *et al.*^45^	To provide a high-quality reference genome for *P. malariae* to improve understanding of this species	NC	NC	*P. malariae, P. ovale*	Uganda, Papua New Guinea, Malaysia, Guinea	Assembly of a high-quality reference genome of *P. malariae* and a manually curated draft *P. o. curtisi* genome providing an important resource to better understand these species.

Abbreviations: NC: not clear; ACT: artemisinin combination therapy; *Pvmdr1*: *P. vivax*, multidrug resistance 1; *Pvcrt-o*: *P. vivax* putative transporter protein; *Pvdhfr*: *P. vivax* dihydrofolate reductase; *Pvdhps:*  *P. vivax* dihydropteroate synthetase.

^a^The number of cases samples obtained from is unclear.

## Discussion

### Malaria epidemiology

Our study highlights key trends in imported malaria to Australia over the last decade which mirror global patterns.^3^ Between 2012 and 2015, case numbers decreased by 35%, but then rose again until 2019. While most reported cases were *P. falciparum* and acquired in Sub-Saharan Africa, the proportion of cases acquired in Oceania and attributable to *P. vivax* was notably higher than that reported from most other non-endemic countries (Supplementary material, Table S3), where *P. vivax* only accounts for 4–19% of cases and 5% or fewer of cases are acquired in Oceania.^14^^,^^46–49^

Imported malaria poses an ongoing threat to malaria-free countries, since travellers and migrants can reintroduce infections in areas with competent mosquito vectors.^14^^,^^15^ Despite increasing trends in international travel and migration to Australia,^38^^,^^50^^,^^51^ malaria incidence in travellers has decreased since 2005.^36^ These trends contrast with other non-endemic countries such as the USA, where travel-related cases increased prior to the COVID-19 pandemic,^52^ and European countries where annual case numbers have remained largely stable from 2010 to 2019.^53–55^

During 2020–2021, COVID-19-related travel restrictions led to a substantial reduction in travel, particularly short-term holiday and business trips. This resulted in a proportionally larger number of migrant arrivals and expatriate returns from malaria-endemic countries, groups inherently at a higher risk of malaria acquisition. Additionally, travellers returning during this period were subjected to mandatory quarantine and closely monitored for fever, which would have prompted COVID-19 testing and investigations for other diagnoses, including malaria, potentially leading to increased detection of sub-clinical or asymptomatic malaria infections.

In Australia, *P. vivax* accounted for almost one in three imported cases, contrasting with other non-endemic countries, where *P. falciparum* causes by far the greatest burden of disease,^14^^,^^46–48^^,^^56–59^ likely reflecting Australia’s geographic location, migration patterns and travellers’ destinations to Asia Pacific where *P. vivax* is the most common malaria parasite.

Africa (most commonly Sudan, Uganda and Nigeria) was an important source of imported cases, likely reflecting increased immigration from this region,^51^ with infections detected both on arrival and following VFR travel.^60^ VFR travellers tend to be less inclined to seek, and less successful in adhering to, mosquito avoidance measures and malaria chemoprophylaxis compared with other traveller groups,^61^^,^^62^ thereby increasing infection risk. Most *P. falciparum, P. ovale* and *P. malariae* cases in our study were acquired in Africa, consistent with global species distribution.^63^^,^^64^ However, we found a lower proportion of imported cases acquired in Africa compared with European and North American studies (68–95%),^14^^,^^15^^,^^46^^,^^47^^,^^56^^,^^58^ reflective of traveller patterns. A study from New Zealand, which has a similar traveller profile to Australia, demonstrated that PNG and the Western Pacific accounted for the majority of imported malaria cases (39%), with Africa accounting for 21%.^60^

Oceania, particularly PNG, was the second highest acquisition region, reflecting its high disease burden and exposure risk.^3^^,^^65^ Malaria is endemic in PNG, the Solomon Islands and Vanuatu, with varying degrees of transmission intensity; PNG has the highest burden (87% of cases in the WHO Western Pacific region in 2021).^3^ In our study, most cases originating from Oceania were *P. vivax* (67%), aligning with *Plasmodium* species distribution in the Western Pacific region where the proportion of *P. vivax* infections has increased from 17% in 2000 to almost one-third in 2021.^3^ Despite global efforts at control, the trajectory of malaria is increasing in PNG and the Solomon Islands along with an increase in *P. vivax* infections. Limited molecular surveillance in these countries highlights the value of Australian data.

Despite remaining notable regions for malaria acquisition, imported cases from Southern and Central Asia declined during the study period. India contributed the majority of cases, reflecting migration and travel trends.^15^ Almost 3% of the Australian population reports India as their birth country^66^ and it is a popular travel destination,^38^ particularly for VFR travellers.^67^ Although the proportion of reported malaria cases due to *P. falciparum* in India has increased from 12% in 1976 to 67% in 2015,^68^ India (especially urban areas) still has the greatest *P. vivax* burden in the region.^69^ Our finding that over 90% of imported cases from India were due to *P. vivax* is likely attributable to travellers more frequently visiting urban areas and that malaria chemoprophylaxis options typically prescribed in Australia (doxycycline and atovaquone-proguanil) do not prevent against *P. vivax* relapses. These findings have important implications for chemoprophylaxis and treatment of malaria in travellers to and from this region.

Despite its popularity as a travel destination for Australians, Southeast Asia accounted for only a small proportion of imported cases, with cases declining since 2012, a likely reflection of very low-risk for malaria acquisition following successful control programmes.^3^

While analysis and surveillance of molecular data for imported malaria cases is not yet routine in Australia, innovative research and computational tools are available and studies conducted to date have provided vital insights into clinically relevant mutations and deletions. The Australian-led vivax Genomic Epidemiology Network (vivaxGEN) has identified genetic barcodes (small panels of single nucleotide polymorphisms) and devised an online data analysis tool (vivaxGEN-GEO) that enables researchers, malaria control programmes and other stakeholders to detect imported *P. vivax* cases and to determine their probable country of origin.^70^ Similar tools are in development to detect and classify imported *P. falciparum* infections, with streamlined data processing and results output aiding users with limited bioinformatics expertise (*Barry et al.* unpublished). Substantial efforts are ongoing to share these resources with malaria-endemic countries.

Imported malaria studies can provide valuable insights into malaria epidemiology and serve as early warning systems potentially informing further research and policy changes, such as by offering insights into parasite mutations, including those that lead to the emergence and spread of drug resistance in countries of acquisition before local detection. For example, the detection of chloroquine resistance-associated mutations in imported *P. vivax* cases from regions of Asia and Oceania,^42^ where chloroquine was still the treatment of choice for *P. vivax* infections in many countries, has led to further studies being conducted in the region, identifying the increasing scale of the problem.^71^ The detection of gene mutations associated with *P. falciparum* artemisinin resistance in imported cases from PNG and surrounding regions^21^^,^^41^ highlights the risk of imported resistant *P. falciparum* to Australia and emphasizes the need for clinicians to be aware of potential treatment failures with ACT. As PNG remains the largest source of imported infections to Australia, molecular surveillance could enhance efforts by PNG to identify the scale of parasite resistance, inform national malaria control programmes and treatment policies, and potentially provide an important source of artemisinin resistant parasites for further studies. The detection of pfhrp2 deletions linked to false negative results from some RDTs in imported cases from some African countries^40^ has prompted these countries to plan or conduct subsequent large-scale surveys.^72^^,^^73^

Investigating recurrences in a malaria-free environment has offered unique insights into blood-stage treatment failures (recrudescence) and relapses without confounding by reinfection. Findings from molecular analyses of an imported case of *P. malariae* with clinical recrudescence following directly observed artemether-lumefantrine therapy^43^ suggest that a 3-day artemether-lumefantrine regimen may not be optimal for *P. malariae* due to its long asexual life cycle and emphasize the need for optimized treatment strategies for non-*falciparum* species as malaria control programmes intensify.^74^

Studies conducted on imported malaria cases in Australia also highlight the capacity to establish biobanks of cryopreserved parasite lines that provide higher-quantity and quality genetic material than can be obtained from dried blood spots taken in endemic countries. The *P. vivax* P01 (*PvP01*) and *P. malariae* UG01 (*PmUG01*) reference genomes were established from an imported infections to Australia and offer^44^ unique insights into the biology, epidemiology and evolution of these species.^44^^,^^45^

Australian laboratories are leading studies of malaria molecular surveillance in endemic countries^34^^,^^35^^,^^75–78^ and have applied this expertise to cases from returning travellers for a range of use cases as described. Laboratory and bioinformatics capacity for malaria molecular research in Australia enables further insights to be gained using travellers as a sentinel population (Supplementary material, Table S4). Additionally, Australia has over 40 malaria research labs, many of which have the capacity to conduct drug resistance screening and genetic engineering experiments for supporting the establishment of a biobank of parasite isolates for further research and for contributing to global efforts to control malaria.^79^

Limitations of our study include incomplete data on some variables such as region of acquisition and characterization of *Plasmodium* species. Other variables of interest such as immigrant status, pre-travel healthcare, chemoprophylaxis, purpose and duration of travel and clinical data are not systematically collected by the NNDSS. Data captured in the NNDSS are dynamic and subject to retrospective revisions; therefore, the presented data represent a point-in-time analysis of malaria case notifications and may vary from data reported in published NNDSS and jurisdictional reports covering the same period. Australian travellers diagnosed with malaria while overseas would have been missed in the reported surveillance data. Currently, only a subset of imported malaria cases undergo molecular testing in Australia. The included molecular studies do not encompass all cases within our NNDSS dataset, and some cases with molecular data fall outside our study period. There is also potential for selection bias in the included molecular studies.

Despite these limitations the results are based on a large national dataset, thus enabling observation of trends over time.

## Conclusion

The epidemiology of imported malaria in Australia is shaped by its unique geographic location, traveller demographics, destination preferences and migrant source countries. Notable differences in the region of acquisition and species composition of imported malaria cases, in comparison with patterns seen in Europe and North America, emphasize the vital role of Australian data in enhancing global malaria surveillance efforts. Harnessing the wealth of resources and expertise found in high-income countries like Australia offers the potential to bolster surveillance efforts in high-risk countries where national molecular surveillance systems are unavailable or developing.

Our study highlights the need and opportunities for collaboration among clinicians, public health practitioners, epidemiologists, scientists and researchers actively engaged in malaria molecular surveillance and research in Australia. We strongly advocate for the establishment and funding of a national imported malaria surveillance network to provide a regional perspective and link in with global malaria surveillance efforts. Such a network would not only fortify Australia’s health security but would help bridge critical gaps in molecular surveillance and make a substantial contribution to the global fight against malaria.

## Funding

A.S. is supported by a National Health and Medical Research Council (NHMRC) Postgraduate Scholarship (APP2002792). K.L. is supported by an NHMRC Senior Research Fellowship (GNT1155005). C.L. (GNT1158469) and S.M. (GNT2017229) are supported by NHMRC Investigator Grants.

## Authors’ contributions

A.S. was involved in the development of the study design, conducted the data collection for the epidemiology aspect of the study, contributed to analysis and interpretation, performed the literature search for the epidemiology aspect of the study, wrote the manuscript and developed the tables and figures. A.B. performed the literature search for the molecular/genomics aspect of the study, contributed to writing and editing the molecular/genomics aspect of the manuscript and was involved in the development of Figure 3 and Table 2. S.A. and Q.C. contributed to the literature search for the molecular/genomics aspect of the study and to writing and editing the molecular/genomics aspect of the manuscript. C.L. was involved in the development of the study design, contributed to data interpretation and reviewing and editing of the manuscript. R.L., R.P. and L.F.K. contributed to reviewing and editing the manuscript. P.B. was involved in the development of Figure 3. S.M. was involved in the development of the study design, data interpretation and contributed to the writing and editing of the manuscript. K.L. was involved in the development of the study design, data interpretation and contributed to the reviewing and editing of the manuscript.

## Conflict of interest

None declared.

## Data availability

The datasets generated and/or analysed during the current study are not publicly available due to the restrictions imposed by the ethics approval from the NNDSS and CDNA. These restrictions prevent the authors from sharing datasets provided by the NNDSS unless written approval is granted by the MUHREC, NNDSS and CDNA. However, much of the data, including annual notification numbers, age groups, sex and jurisdiction information, are accessible via the National Notifiable Disease Surveillance System dashboard, found at https://nindss.health.gov.au/pbi-dashboard/. The corresponding authors can be contacted if access to the complete NNDSS dataset utilized in our study is required, but enabling access would necessitate obtaining additional approvals from MUHREC, NNDSS and CDNA.

## Supplementary Material

Supplementary_file_JTM_final_taad164
