# Supplementary material for: Imported malaria into Australia: surveillance insights and opportunities
Source: J Travel Med. 2023 Dec 21;31(3):taad164. doi: 10.1093/jtm/taad164 (PMC10998534; doi:10.1093/jtm/taad164)
Supplement: Supplementary_file_JTM_final_taad164 [file supplementary_file_jtm_final_taad164.docx]

**Supplementary materials**

**Methods**

**Malaria cases:** Variables obtained from the National Notifiable Disease Surveillance System (NNDSS) included disease diagnosis, date of notification, state, age group, gender, country of acquisition and *Plasmodium* species.

**Geographical regions classified according to the Standard Australian Classification of Countries (SACC) 2016:** Oceania and Antarctica, Northwest Europe, Southern and Eastern Europe, North Africa and the Middle East, Southeast Asia, Northeast Asia, Southern and Central Asia, Americas and Sub-Saharan Africa. Sudan and South Sudan were grouped under North Africa and the Middle East region.

**Literature search on malaria surveillance in returned travellers**

A search of electronic databases PubMed and Google Scholar and a standard Google search for “imported malaria”, “malaria in returned travellers” and “malaria in non-endemic countries” was performed. These searches identified academic papers and reports documenting imported cases. Data on country, time period, number of malaria cases, gender, *Plasmodium* species and region of exposure were extracted where available. Notable studies from various countries were compiled into a table (table S3).

**Literature search on malaria genetics and genomics**

As proof of concept on the utility and capacity of conducting genetic and genomic analyses of imported malaria infections in Australia, a search of electronic databases PubMed and Google Scholar for “malaria genomics”, “malaria genetics”, “molecular malaria surveillance”, “*Plasmodium* genomics”, “*Plasmodium* genetics”, “imported malaria surveillance” and “Australia” was performed (limited to the last twenty years). These searches identified academic papers and reports that describe molecular data on imported malaria cases in Australia. A selection of studies that highlight a range of different cases to inform on travel medicine in Australia, surveillance in the countries of origin, and broader malaria molecular research and development are presented. To capture information on the capacity for genetic and genomic processing of imported malaria cases, details were collected from each of the co-authors on the available laboratory equipment and computational resources to conduct targeted genotyping or whole genome sequencing of malaria parasites.

**Results**

**Table S1: Malaria notification incidence by traveller movements, Australia 2012 - 2022**

| **Year** | **Malaria notifications (n)** | **Traveller movements**  **(n)** | **Incidence per 100,000 traveller movements [95% CI]** |
| --- | --- | --- | --- |
| 2012 | 345 | 15,120,660 | 2.28 [2.05-2.54] |
| 2013 | 422 | 15,946,240 | 2.65 [2.40-2.91] |
| 2014 | 324 | 16,772,840 | 1.93 [1.73-2.15] |
| 2015 | 235 | 17,580,460 | 1.34 [1.17-1.52] |
| 2016 | 305 | 18,989,710 | 1.61 [1.43-1.80] |
| 2017 | 364 | 20,123,790 | 1.81 [1.63-2.00] |
| 2018 | 407 | 21,139,640 | 1.93 [1.74-2.12] |
| 2019 | 381 | 21,618,790 | 1.76 [1.59-1.95] |
| 2020 | 157 | 4,949,420 | 3.17 [2.70-3.71] |
| 2021 | 59 | 765,060 | 7.71 [5.87-9.95] |
| 2022 | 205 | 2,866,040 | 7.15 [6.21-8.20] |
| Total | 3,204 | 155,872,650 |  |

95% CI: 95% confidence interval

**Table S2: Ten most common countries of acquisition by jurisdiction, 2012 – 2022**


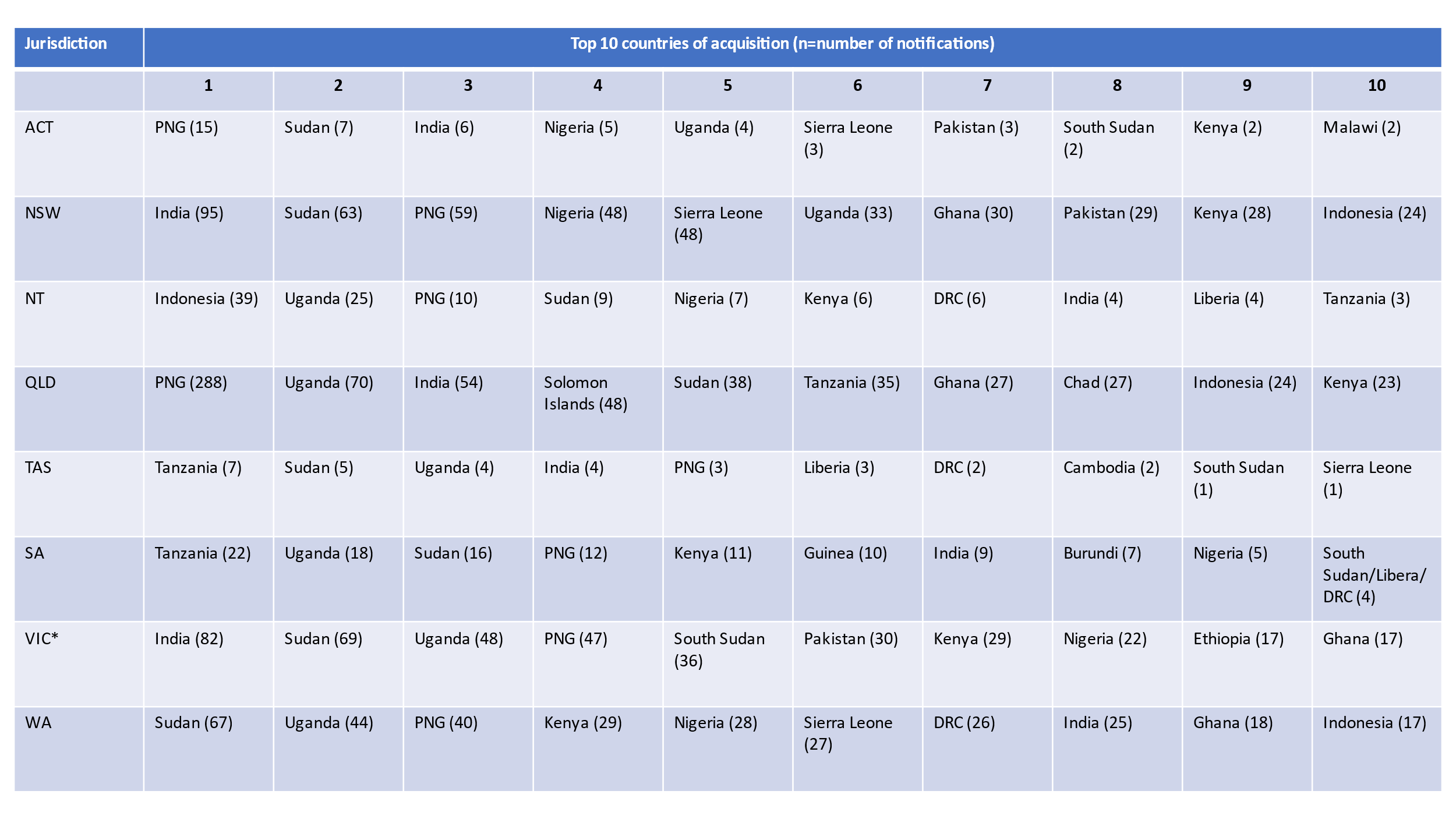


Abbreviations: ACT: Australian Capital Territory; NSW: New South Wales; NT: Northern Territory; QLD: Queensland; TAS: Tasmania; SA: South Australia; VIC: Victoria; WA: Western Australia; PNG: Papua New Guinea; DRC: Democratic Republic of the Congo,

*Country unknown/no data available, n=158

**Figure S1: Malaria notifications for individual regions of acquisition by year, 2012 - 2022**

Other includes Northeast Asia, Northwest Europe, Southern and Eastern Europe, Americas

Sudan and South Sudan are classified under North Africa and the Middle East

**Table S3: Malaria surveillance data in returned travellers to non-endemic countries**

| **Author** | **Study region/ country** | **Data source** | **Time period** | **Total cases, n** | **Male, n (%)** | ***P. falciparum*, n (%)^a^** | ***P. vivax*, n (%)^a^** | **Mixed *Plasmodium* infections** | **Region of exposure, n (%)** | | | | |
| --- | --- | --- | --- | --- | --- | --- | --- | --- | --- | --- | --- | --- | --- |
|  |  |  |  |  |  |  |  |  | **Sub-Saharan Africa** | **South Central Asia** | **Southeast Asia** | **Oceania** | **Other^b^** |
| Angelo KM et al. ^(1)^ | Global | GeoSentinel^c^ | Jan 2003 – Jul 2016 | 5,689 | 3,918 (69) | 4,011 (71) | 857 (15) | 66 (11) | 4,705 (83) | 309 (5) | 266 (5) | 96 (2) | 313 (5) |
| Grobusch MP et al. ^(2)^ | Europe | EuroTravNet^d^ | Mar 1998 – Mar 2018 | 7,195 | NR | 5,254 (73) | NR | 96 (1) | 6,370 (89) | NR | NR | NR | NR |
| Giannone B et al. ^(3)^ | Switzerland | Swiss Federal Office of Public Health | 1990-2019 | 8,439 | 5,280 (63) | 5,102 (61) | 1,554 (18) | 129 (1.5) | Africa 5,715 (68) | Asia 493 (6) | NR | 48 (<1) | 191 (2) |
| Lu G et al. ^(4)e^ | China | China Centres for Disease Control and Prevention | Jun 2012 – Dec 2019 | 2,255 | 2,194 (97) | 1,769 (78) | 113 (5) | 13 (<1) | 1,768 (78) | NR | NR | NR | NR |
| Norman et al.^(5)^ | Spain | +REDIVI Network^f^ | Oct 2009 – Oct 2019 | 850 | 483 (57) | 693 (82) | 34 (4) | 22 (3) | 809 (95) | Asia 21 (2) | NR | NR | 20 (2) |
| De Gier et al. ^(6)^ | Netherlands | Osiris^g^ | Jan 2008 – Mar 2016 | 1,971 | NR | 1,337 (68) | 372 (19) | 13 (<1) | 1,468 (74) | NR | NR | NR | NR |
| Kaftandjiev I et al. ^(7)^ | Bulgaria | Clinical records/epidemiological survey | 2000 – 2020 | 232 | 125 (85) | 150 (65) | 66 (28) | 4 (2) | 169 (73) | Asia 61 (26) | NR | NR | 2 (<1) |
| Kanayama et al. ^(8)^ | Japan | NESID^h^ | 2006 - 2014 | 557 | 425 (76) | 183 (33) | 78 (14) | NR | 293 (53) | Asia 126 (23) | NR | 28 (5) | NR |
| Kendjo E et al. ^(9)^ |  | French National Reference Center of Malaria Surveillance | 1996 – 2016 | 43,333 | 24,949 (62) | 37,065 (86) | 1,732 (4) | 736 (2) | 41,780 (96) | NR | NR | NR | NR |
| Rees E et al. ^(10)^ | France | PHE MRL^i^ | 2000 – 2014 | 15,473 | 9,177 (59) | 12,713 (82) | 1,497 (10) | NR | 6,404 (41) | 394 (3) | NR | NR | NR |
| This series | Australia | NNDSS^j^ (Australia) | Jan 2012 – Dec 2022 | 3,204 | 2,196 (68) | 1,840 (57) | 996 (31) | 34 (1) | 1,433 (45) | 367 (12) | 194 (6) | 569 (18) | 375 (12) |

Abbreviations: NR: Not reported; REDIVI: Red Cooperativa para el Estudio de Enfermedades Importadas por Viajeros e Inmigrantes; NESID: National Epidemiological Surveillance of Infectious Disease System; PHE MRL: Public Health England Malaria Reference Laboratory; NNDSS: National Notifiable Disease Surveillance System; Dec: December; Jan: January; Jul: July; Jun: June; Mar: March; Oct: October

^a^Excludes mixed *Plasmodium* spp. infections

^b^Includes cases from the following regions: Northeast Asia, Northwest Europe, Southeast Europe, North Africa/Middle East, Americas. Missing data not included

^c^A global surveillance network of travel and tropical medicine providers, <https://geosentinel.org/>

^d^European surveillance sub-network of GeoSentinel, therefore some overlap with cases reported in Angelo KM et al.

^e^Study conducted in Jiangsu Province, China which has had no endemic cases since 2012

^f^The Spanish Network for the Study of Infectious Diseases imported by Travellers and Immigrants (Red Cooperativa para el Estudio de Enfermedades Importadas por Viajeros e Inmigrantes)

^g^The Dutch Electronic National Surveillance System, The Netherlands

^h^National Epidemiological Surveillance of Infectious Disease System, Japan

^i^Public Health England Malaria Reference Laboratory, England

^j^The National Notifiable Disease Surveillance System, Australia

**Table S4: Laboratory and computing capacity for molecular analysis of malaria parasites in selected Australian institutes**

| **State/Territory** | **Institute(s)** | **Targeted genotyping capacity** | **Whole genome sequencing capacity** | ***HPC capacity** |
| --- | --- | --- | --- | --- |
| Northern Territory | Menzies School of Health Research | Microsatellite genotyping;  Illumina amplicon sequencing;  MinION amplicon sequencing | Illumina;  MinION | HPC with 8 computing nodes (32 Dualcores CPU each (AMD), 1 GPU node (2 x 32GB GPUs (Nvidia) ) and 224TB raw storage |
| Queensland | ADFMIDI | Multiplex qPCR and multiplex digital PCR for detecting *pfhrp2/3* deletions;  Sanger sequencing;  Microsatellite genotyping;  Developing Illumina and MinION amplicon sequencing | Currently rely on AGRF for WGS; Developing Illumina and MinION | Project based collaboration with the University of Queensland |
| Victoria | Burnet Institute;  Deakin University | qPCR (SNP genotyping and copy number variants)  microsatellite genotyping, Saner sequencing;  Illumina and MinION amplicon sequencing | Illumina;  MinION;  Ion Torrent | National Computing Infrastructure: HPC with 4,962 nodes, more than 250,000 CPU cores, 930 Terabytes of memory and 640 GPUs comprising Intel Sapphire Rapids, Cascade Lake, Skylake and Boardwell CPUs and NVIDIA V100 and DGX A100 GPUS. We have ongoing access to the National Computing Infrastructure (NCI) Australia which includes 40kSU every quarter and 6 TB of data storage.  Deakin University: HPC Cluster includes XENON HPC, 4 main nodes including compute node (CPU capacity), GPU node (3 DGX machines 8 NVIDIA A100 cards), FAT node (access to 1TB memory) and VDI Node (virtual desktop node using T4 NVIDIA GPU). |

Abbreviations: HPC: high performance computer; ADFMIDI: Australian Defence Force Malaria and Infectious Disease Institute; qPCR: quantitative polymerase chain reaction; AGRF: Australian Genomic Research Facility; WGS: whole genome sequencing; SNP: single nucleotide polymorphism

**References**

1. Angelo KM, Libman M, Caumes E, Hamer DH, Kain KC, Leder K, et al. Malaria after international travel: a GeoSentinel analysis, 2003-2016. Malar J. 2017;16(1):293.

2. Grobusch MP, Weld L, Goorhuis A, Hamer DH, Schunk M, Jordan S, et al. Travel-related infections presenting in Europe: A 20-year analysis of EuroTravNet surveillance data. Lancet Reg Health Eur. 2021;1:100001.

3. Giannone B, Hedrich N, Schlagenhauf P. Imported malaria in Switzerland, (1990–2019): A retrospective analysis. Travel Medicine and Infectious Disease. 2022;45:102251.

4. Lu GA, Cao Y, Chen Q, Zhu G, Müller O, Cao J. Care-seeking delay of imported malaria to China: implications for improving post-travel healthcare for migrant workers. J Travel Med. 2022; 29(4): taab156.

5. Norman FF, López-Polín A, Salvador F, Treviño B, Calabuig E, Torrús D, et al. Imported malaria in Spain (2009-2016): results from the +REDIVI Collaborative Network. Malar J. 2017; 16(10): 407.

6. de Gier B, Suryapranata FS, Croughs M, van Genderen PJ, Keuter M, Visser LG, et al. Increase in imported malaria in the Netherlands in asylum seekers and VFR travellers. Malar J. 2017; 16(1): 60.

7. Kaftandjiev I, Harizanov R, Rainova I, Mikov O, Tsvetkova N, Borisova R, et al. Epidemiological and clinical characteristics of imported malaria in Bulgaria: A retrospective study of а 21-year period. Travel Medicine and Infectious Disease. 2022;49:102400.

8. Kanayama A, Arima Y, Matsui T, Kaku K, Kinoshita H, Oishi K. Epidemiology of Imported Malaria Cases in Japan, 2006-2014: A Sentinel Traveler Surveillance Approach. Am J Trop Med Hyg. 2017; 97(5): 1532-1539.

9. Kendjo E, Houzé S, Mouri O, Taieb A, Gay F, Jauréguiberry S, et al. Epidemiologic Trends in Malaria Incidence Among Travelers Returning to Metropolitan France, 1996-2016. JAMA Network Open. 2019;2(4):e191691-e.

10. Rees E, Saavedra-Campos M, Usdin M, Anderson C, Freedman J, de Burgh J, et al. Trend analysis of imported malaria in London; observational study 2000 to 2014. Travel Med Infect Dis. 2017;17:35-42.
